# Serum selenium levels do not differ in type 2 diabetic subjects with and without coronary artery disease

**DOI:** 10.1186/1756-0500-4-270

**Published:** 2011-07-29

**Authors:** Alexios Sotiropoulos, Stavroula A Papadodima, Athanasia K Papazafiropoulou, Aggelos Ioannidis, Athanasia Kokkinari, Ourania Apostolou, Chara A Spiliopoulou, Sotirios Athanaselis

**Affiliations:** 1 3rd Department of Internal Medicine and Center of Diabetes, General Hospital of Nikaia "Ag. Panteleimon" - Piraeus, Greece; 2 Department of Forensic Medicine and Toxicology, National and Kapodistrian University of Athens, Greece

**Keywords:** selenium, type 2 diabetes, cardiovascular disease

## Abstract

**Background:**

The aim of the present study was to investigate whether selenium levels differ between type 2 diabetic subjects with and without coronary artery disease (CAD).

**Methods:**

A total of 200 subjects with type 2 diabetes (100 with CAD and 100 without CAD), consecutively selected from the diabetes outpatient clinic of our hospital were enrolled into the study. A detailed medical history and a physical examination were obtained by all the participants.

**Results:**

Serum selenium levels did not differ between diabetic subjects with and without CAD (102.40 ± 31.10 vs. 108.86 ± 33.88 microg/L, p = 0.16). In diabetic subjects with CAD multivariate linear regression analysis demonstrated significant independent associations between selenium and sex (beta = 0.21, p = 0.03) and glucose levels (beta = 0.25, p = 0.008). In diabetic subjects without CAD multivariate linear regression analysis demonstrated significant independent associations between selenium and peripheral artery disease (beta = 0.16, p = 0.05) and glucose levels (beta = -0.09, p = 0.05).

**Conclusion:**

Serum selenium levels did not differ between diabetic subjects with and without CAD. In diabetic subjects with CAD, the only determinants of serum selenium levels were sex and glucose levels. In diabetic subjects without CAD the only determinants of serum selenium levels were peripheral artery disease and glucose levels.

## Background

Studies have showed that selenium is an essential trace element mainly involved in the complex system of defense against oxidative stress [1,2], thyroid function and immune functions [3]. Despite the above functions, studies have showed a potential association between serum selemium levels and cardiovascular risk [4-7]. High selenium levels have been associated with higher prevalence of hypertension [7]. However, the existing literature data are still inconsistent [4-7]. Despite the reports showing association between low serum selenium levels and increased cardiovascular mortality and morbidity, the protective role of selenium against cardiovascular diseases still remains debated [3].

The existing literature data regarding the relationship between serum selenium levels and type 2 diabetes (T2D) are controversial [8-13]. Some studies showed lower serum selenium levels in diabetic subjects compared with nondiabetic subjects [8,9]. Experimental data showed that selenium might play a role to the regulation of specific beta-cell target genes and potentially promotes an overall improvement in islet function [10]. On the other hand, it has been showed that high serum selenium levels were positively associated with the prevalence of diabetes [11]. In addition, serum selenium levels have been associated with the onset of T2D. It was hypothesized that high levels of selenium might prevent diabetes [12]. However, recent studies found that selenium supplementation did not prevent T2D, and it may increase the risk for the disease [13,14].

Since the existing literature data regarding the role of serum selenium levels to diabetes and its complications are controversial we conducted the present study in order to investigate whether selenium levels differ between T2D subjects with and without coronary artery disease (CAD).

## Methods

### Subjects and procedures

A total of 100 type 2 diabetic subjects with CAD and 100 diabetic subjects without CAD, consecutively selected from the diabetes outpatient clinic of our hospital were enrolled into the study from December 2009 to May 2010. Diagnosis of diabetes was based on the American Diabetes Association criteria [15]. They were questioned about previous and current diseases, use of medications and their smoking habits, vitamin use, and a thorough physical examination was performed. Subjects were considered as non-smokers if they have never smoked or if they had given up smoking for at least three consecutive years. Subjects that had received selenium supplements were excluded by the study. Diet was assessed using a semi-quantitative food-frequency questionnaire designed to capture dietary habits [16]. The nutritional database maintained by the Italian National Institute of Nutrition was used to report the average selenium content of several foodstuffs [17].

All measurements were performed in the morning, after 10-12 hours fast. The subjects were advised not to eat, smoke, or drink coffee before examination. Blood samples were drawn for measurement of plasma glucose, HbA_1c_, creatinine, and lipid profile. The antidiabetic medications were given to the patients at the end of the examination. Blood pressure was measured three consecutive times, one minute in apart, in the sitting position using an appropriate cuff size. The mean values of the last 2 measurements was calculated and used in the analysis. Arterial hypertension was defined according to the current guidelines [18], when systolic was ≥ 140 mmHg or and/or diastolic blood pressure was ≥ 90 mmHg or when the patients were on antihypertensive treatment. CAD was defined as presence of angina, history of previous myocardial infarction, positive stress testing, revascularization procedures or stenosis > 50% at the coronary arteries. Direct fundoscopy was performed in all subjects through dilated pupils.

Body weight with subjects in light clothing without shoes and height was measured and body mass index (BMI) was calculated. Waist circumference was measured with a soft tape on standing, midway between the lowest rib and the iliac crest.

The purpose of the study was clearly explained to all subjects, who then volunteered to participate. The ethics committee of the General Hospital of Nikaia approved the study and informed written consent was obtained.

### Analytical methods

Fasting serum glucose, lipids and creatinine concentrations were measured on a Technicon analyzer RA-XT. Low density lipoprotein (LDL) cholesterol concentrations were calculated using the Friedewald formula [19]. HbA_1c _was measured by high-performance liquid chromatography (HPLC) (Roche Diagnostics, Mannheim, Germany) with a non-diabetic reference range of 4.0-6.0%. Microalbuminuria was assessed by measuring the albumin-to-creatinine ratio (ACR) in a random urine sample on a DCA 2000 analyzer using the immunonephelometry technique (Bayer HealthCare LLC, Elkhart, USA). Appropriate trace-element tubes (Becton-Dickinson, Vacutainer; NJ, USA) were used for drawing blood samples for determination of selenium levels in blood. Serum levels of selenium were determined by use of atomic mass spectrometry (ZEEnit 700; Analytical Jena, Germany).

### Statistical analysis

Statistical analysis was preformed using programs available in the SPSS statistical package (SPSS 10.0, Chicago, USA). All variables were tested for normal distribution of the data. Data are shown as mean ± SD, unless it is stated otherwise. A two sample *t*-test was used to assess differences in continuous variables, while a chi-square test was used for categorical variables. Univariate linear regression analysis was performed to look for the relationship between serum selenium and the variables of interest in the sample population. Then, multivariate linear regression analyses were performed (backward stepwise method) to look for independent associations between serum selenium and the variables of interest. All independent variables in the multivariate analyses models were tested for multicolinearity. p < 0.05 (two-tailed) was considered statistically significant.

## Results

Serum selenium levels did not differ between diabetic subjects with and without CAD (102.40 ± 31.10 vs. 108.86 ± 33.88 microg/L, p = 0.16).

The demographic and clinical characteristics of the two study groups are showed in Table 1. Of the diabetic subjects with CAD 30.8% was on treatment with sulphonylurea, 59.8% on metformin, 3.7% on glitazones, 3.7% on acarbose, 2.8% on megltitinides, 8.4% on DPP-4 inhibitors, and 51.5% on insulin. Of the diabetic subjects without CAD 47.0% was on treatment with sulphonylurea, 69.7% on metformin, 13.6% on glitazones, 3.0% on acarbose, 1.5% on megltitinides, 4.5% on DPP-4 inhibitors, and 44.6% on insulin. Statin use did not differ between the two study groups (Table 1). Diabetic subjects without CAD had higher values of systolic and diastolic blood pressure compared to diabetic subjects with CAD. Diabetic subjects with CAD had more often retinopathy and lower serum levels of total and LDL-cholesterol than diabetics without CAD. Diabetic subjects without CAD used more often vitamins of the B and C complex than those with CAD. In addition, diabetic subjects with CAD were more often smokers than diabetic subjects without CAD (Table 1).

**Table 1:** Demographic and clinical characteristics of diabetic subjects according to the presence (CAD+) or not (CAD-) of coronary artery disease

	CAD (-)	CAD (+)	*P*
Males/females (%)	52/48	52/48	-
Age (years)	66.68 ± 9.52	65.60 ± 8.16	0.38
Body mass index (kg/m2)	32.66 ± 5.20	33.20 ± 5.53	0.48
Waist (cm)	104.31 ± 12.51	106.62 ± 12.10	0.18
Systolic blood pressure (mm Hg)	148.65 ± 19.34	142.75 ± 18.24	**0.02**
Diastolic blood pressure (mm Hg)	80.74 ± 9.68	77.70 ± 9.27	**0.02**
Duration of diabetes (years)	11.83 ± 8.28	13.23 ± 8.27	-
Smoking (yes) (%)	22 (21.8)	35 (35.7)	**0.03**
Alcohol consumption (yes) (%)	39 (38.6)	35 (35.7)	0.67
Vitamins (yes) (%)	15 (15.0)	5 (5.1)	**0.02**
Hypertension (yes) (%)	87 (86.1)	92 (93.4)	0.07
Dyslipidemia (yes) (%)	90 (89.1)	92 (93.4)	0.22
Any retinopathy (yes) (%)	15 (14.9)	27 (27.6)	**0.03**
Neuropathy (yes) (%)	6 (5.9)	9 (9.2)	0.67
Microalbuminuria (yes) (%)	49 (52.7)	58 (61.1)	0.24
Peripheral artery disease (yes) (%)	7 (5.9)	14 (14.3)	0.09
Treatment for diabetes	-	-	-
Antidiabetic tablets (yes) (%)	81 (80.2)	66 (67.3)	**0.04**
Insulin (yes) (%)	45 (44.6)	50 (51.5)	0.32
HbA1c (%)	7.58 ± 1.72	7.74 ± 1.74	0.51
Statins (yes) (%)	89 (67.4)	95 (88.8)	0.15
Glucose (mg/dl)	159.83 ± 53.63	160.74 ± 58.53	0.90
Total cholesterol (mg/dl)	181.56 ± 40.80	168.48 ± 43.85	**0.03**
HDL cholesterol (mg/dl)	46.52 ± 10.99	43.40 ± 10.38	**0.04**
LDL cholesterol (mg/dl)	106.13 ± 37.92	94.26 ± 37.25	**0.03**
Triglycerides (mg/dl)	153.14 ± 82.00	158.08 ± 87.97	0.68
Urea (mg/dl)	44.91 ± 23.34	45.77 ± 21.00	0.78
Creatinine (mg/dl)	1.00 ± 0.55	0.99 ± 0.24	0.88
Uric acid (mg/dl)	5.54 ± 1.61	5.65 ± 1.68	0.64
CRP (mg/dl)	4.32 ± 1.16	3.7 ± 1.8	0.52
Ht (%)	40.16 ± 3.66	40.41 ± 3.55	0.11
WBCs (n)	7,561.68 ± 2,038.28	8,040.91 ± 2,196.38	0.11
PLTs (n)	250,980.20 ± 72,600.54	242,969.07 ± 17,043.47	0.66
Serum Selenium (microg/L)	108.86 ± 33.88	102.40 ± 31.10	0.16
Dietary Selenium (microg/L)	1.04 ± 0.47	1.15 ± 0.65	0.19

P values for the comparison between groups with and without metabolic syndrome (MS) by independent samples t-test for continuous variables or by Pearson χ^2 ^for nominal variables.

HbA_1c_: glycated hemoglobin A1c; HDL: high density lipoprotein; LDL: low density lipoprotein; CRP: high sensitivity C-reactive protein; Ht: Hematocrit; WBCs: white blood cells count; PLTs: platelets.

In diabetic subjects with CAD univariate linear regression analysis showed that selenium levels were associated significantly with gender [standardized regression coefficient (beta) = 0.25, p = 0.009], BMI (beta = -0.22, p = 0.02), glucose levels (beta = 0.21, p = 0.03), number of platelets (beta = -0.19, p = 0.05) and C-reactive protein (beta = -0.19, p = 0.04). Multivariate linear regression analysis demonstrated, after controlling for the above factors, significant independent associations between selenium and gender (beta = 0.21, p = 0.03) and glucose levels (beta = 0.25, p = 0.008).

In diabetic subjects without CAD univariate linear regression analysis showed that selenium levels were associated significantly with systolic blood pressure (beta = 0.22, p = 0.01), peripheral artery disease (beta = 0.15, p = 0.05) and glucose levels (beta = -0.16, p = 0.05). Multivariate linear regression analysis demonstrated significant independent associations between selenium and peripheral artery disease (beta = 0.16, p = 0.05) and glucose levels (beta = -0.09, p = 0.05) (Table 2).

**Table 2:** Multivariate linear regression analysis: the association between various parameters with serum selenium and in diabetic subjects with and without coronary artery disease (CAD)

	*β*	*P*
**Diabetic subjects with CAD (Model 1)**		
Gender (males vs. females)	0.21	0.03
Glucose	0.25	0.008
**Diabetic subjects without CAD (Model 2)**		
Peripheral artery disease (yes vs. no)	0.16	0.05
Glucose	-0.09	0.06

*β*: standardized regression coefficient.

Additional variables tested in model 1: body mass index, platelets and C-reactive protein.

Additional variables tested in model 2: systolic and diastolic blood pressure.

## Discussion

The results of the present study showed that serum selenium levels did not differ between diabetic subjects with and without CAD. In diabetic subjects with CAD, the only determinants of serum selenium levels were sex and glucose levels. In diabetic subjects without CAD the only determinants of serum selenium levels were peripheral artery disease and glucose levels.

The existing literature data regarding serum selenium levels in diabetic and nondiabetic subjects are conflicting [20-24]. In a recent study in Asia there were no important differences for serum selenium levels in T2D subjects and nondiabetic individuals [12]. On the contrary, two studies showed that the mean serum selenium levels in diabetic subjects were significantly lower than in controls [9,21]. In addition, a negative correlation between the plasma contents of selenium and HbA1c was found, confirming our findings regarding the relationship between selenium and glucose levels in both study groups [20]. Other authors found a statistically significant increase in selenium levels in subjects with diabetes to the control group [10,22]. A cross-sectional analysis of 8,876 adults over 20 years of age who participated in the Third National Health and Nutrition Examination Survey in the USA showed that high serum selenium levels were positively associated with the prevalence of diabetes [11]. Serum selenium levels were associated with higher fasting plasma glucose and HbA1c [22]. An observational analysis conducted within the Supplementation with Antioxidant Vitamins and Minerals (SU.VI.MAX) trial, in which baseline plasma selenium levels were positively associated with combined fasting plasma glucose at baseline and after 7.5 years of follow-up [23]. These findings are in contrast with a cross-sectional analysis of the Health Professionals Study, which showed an inverse association between toenail selenium and diabetes prevalence [24].

It is noteworthy that a study in the early 80s showed elevated serum selenium levels in diabetic children compared with healthy counterparts [25]. The authors explained the difference by the altered lipid metabolism [25]. A study in females with gestational diabetes mellitus showed that females with gestational diabetes mellitus and those with glucose intolerants had lower serum selenium levels than that of the normal pregnant women [26]. The same study found a significant inverse correlation between serum selenium and blood glucose levels that is in accordance with our results [26].

A study aiming to evaluate the association between toenail selenium and CAD among men with diabetes showed that levels of selenium were lower among diabetic men with or without CAD than among healthy controls [24]. However, this study could not distinguish between the effects of selenium on diabetes and those on CAD [23]. In healthy subjects, two studies reported a null association between selenium and risk of CAD [27,28]. Finally a recent analysis of the Olivetti Heart Study failed to show possible associations between selenium levels and cardiometabolic risk factors in healthy men [29].

## Limitations

The present study has its limitations. First of all no power sample calculation was performed that could eliminate possible error type II in the study. In addition, the present study as a retrospective one can not clarify if selenium acts as a marker or as a cause of CAD in diabetic subjects. Also, physical activity, energy intake and presence of metabolic syndrome were not examined for possible associations with selenium levels in the two study groups. In addition, other comorbidities correlated with selenium levels as lung cancer and asthma were not examined. Finally, the findings of the present study refer to diabetic population attending an outpatient clinic and, therefore, an etiology hypothesis cannot be confirmed with this study.

## Conclusions

In conclusion, the present study showed that serum selenium levels did not differ between diabetic subjects with and without CAD. These results showed that selenium, despite its anti-oxidatant functions, cannot be used for the prevention of CAD in diabetic subjects. However, further, longitudinal studies are needed in order to evaluate the role of serum selenium levels in T2D and CAD.

## Competing interests

The authors declare that they have no competing interests.

## Authors' contributions

AS, AI, and OA participated in the collection of the data. AS, SP, SA, AP and CS participated in the design of the study and performed the statistical analysis and drafted the manuscript. All authors gave final consent for publication.
